# Exploring barriers to reproductive, maternal, child and neonatal (RMNCH) health-seeking behaviors in Somali region, Ethiopia

**DOI:** 10.1371/journal.pone.0212227

**Published:** 2019-03-15

**Authors:** Moti Tolera Jalu, Abdurehman Ahmed, Abdiwahab Hashi, Alula Tekilu

**Affiliations:** 1 School of Public Health, Haramaya University, Harar, Oromia Region, Ethiopia; 2 Department of Public Health, Debre berihan University, Debre berihan, Amhara Region, Ethiopia; 3 Department of Public Health, Jigjiga University, Jigjiga, Somali Region, Ethiopia; 4 St. Paul’s Millennium Medical College, Addis Ababa, Ethiopia; 5 Monitoring and Evaluation and Research Quality control (MERQ) Consulting PLC., Addis Ababa, Ethiopia; University of Botswana, BOTSWANA

## Abstract

**Introduction:**

Health-seeking behaviours are influenced by internal and external contributing factors. Internal factors include attitudes, beliefs and core values, life adaptation skills, psychological disposition whereas external factors include social support, media, socio-cultural, political, economic and biological aspects, health care systems, environmental stressors and societal laws and regulations. This study was meant to explore factors affecting health-seeking behaviors in the Somali regional state of Ethiopia. The study employed a cross-sectional study design using qualitative data collection tools. Data were collected from 50 individual interviews and 17 focused group discussions (FGD) on women of reproductive age and their partners, health extension workers (HEWs), health care providers and health administrators. To ensure representativeness, the region was categorized into three zones based on their settlement characteristics as agrarian, pastoralist and semi-pastoralist. Two districts (one from high and the other from low performance areas) were selected from each category. The data were entered, coded, categorized and analyzed using NVIVO version 11 software. The Socio-ecologic Model (SEM) was used for categorization.

**Results:**

Using the social ecological model, the following major barriers for health seeking behaviors were identified. Low socio-demographic and economic status, poor exposure to health information or mass media, detrimental preferences of breast feeding methods and short acting family planning (FP) methods were identified barriers at the individual level; male dominance in decision making, the influence of the husband and society and the role of word of mouth were identified barriers at the interpersonal level and lack of acceptance, fear of modern health practices, unclean health facility environment, lack of well-equipped facilities shortage of trained staffs and barriers relating to distance and transportation were barriers identified at organizational and policy level.

**Conclusion:**

Overall, factors at various level affected health seeking behaviors of the Somali community. Socio-demographic and economic factors, non-responsive bureaucratic system, shortages or absence of medical supplies and human resources, lack of supportive supervision, a shortage of water and electricity at the health facility and an unclean service delivery environment are significant barriers to health-seeking behaviors for the community.

## Introduction

### Background

Health-seeking behaviour is influenced by internal and external factors. Internal factors are attitudes, beliefs and core values, life adaptation skills, psychological disposition. External factors are social supports, media, socio-cultural, political, economic and biological aspects, health care systems, environmental stressors and the laws and regulations of society [1].

Studies have reported that there are residence-based difference in using health services as urban women have more access to education than those who live in rural areas, whereas rural women have a higher chance of a high risk pregnancy [2, 3]. Moreover, women’s education plays a vital role in their economic, socio-cultural and political empowerment [3, 4]. Additionally, high educational status is directly related to economic and social empowerment of women which then increases her chance of accessing the media and utilizing the required health care services as needed [5].

Most rural women who are uneducated and poor and are less likely to use facility services, furthermore, a woman who have previous use of facility services have higher chance to the consecutive services she needs in the continuum of care. For example, a women who use ANC services have higher chance to use delivery care services [3].

Interpersonal and social factors like religion, culture peer pressure, and organizational factors like access and trust on health care providers can also negatively affected the health seeking behaviour of the community and an individual as well [2, 6–11].

Globally Ten million women are estimated to develop difficulties related with pregnancy each year, with half a million of these dying as a result where almost all death (99%) of deaths occur in developing countries [12]. There is also inequity in service utilization among poor and rich countries with fewer than 10% of births in poor communities use skilled birth attendant, compared to 56% of births for mothers from the richest income quintile in that region [13].

Ethiopia is one of the four countries with the highest number of maternal deaths [14]. Only 15% of deliveries in Ethiopia are attended by a skilled provider [15], compared to 51% for other African regions [16]. Similarly the poorest households had greater maternal mortality rate (MMR) and neonatal mortality compared to richest (550 versus 239 per 100,000 live births) [17].

Improving maternal health remains a top priority of the Ethiopian Federal Ministry of Health (FMoH) as outlined in the health sector transformation plan (HSTP) [18]. The maternal death surveillance report (MDSR) program is a fundamental component of the FMoH’s efforts to reduce maternal mortality, along with the use of Health Extension Workers (HEW) in promotion of antenatal care and safe delivery services at the place of choice for mothers [18–20]. However, in line with a movement towards decentralisation of policy-making, the specific tasks and responsibilities of each HEW is largely determined at the regional and sometimes district levels [21, 22].

Many studies have shown that the health-seeking behaviour and service utilizations of Ethiopia is poor compared to other African regions. Evidences also indicated that Somali regional state’s health seeking behaviour and service utilizations are the worst health indicators nationally. Somali region has the second highest teenage pregnancy (22%) next to Afar, the second lowest immunisation rates (48%) next to Afar, the Contraceptive Prevalence Rate (CPR) is the lowest (2%), Antenatal Care (ANC) is the lowest with 12% of ANC4^th^ and the highest percentage (23%) of children with wasting [15, 23]. These statistics make it imperative to learn about the barriers to healthcare seeking among households across different neighbourhoods in the regional state in order to contribute to the development of community-responsive health policies. Whereas previous research has highlighted the poor health-seeking behaviour in Ethiopia generally no other literature has qualitatively explored and described the barriers in health seeking behaviour of the communities in the Somali region.

### Objectives of the study

**General objective**

- To explore barriers influencing health care seeking behaviours in the Somali regional state of Ethiopia.

**The specific objectives**

- To identify barriers to reproductive health care-seeking behaviours.
- To identify barriers to maternal health care-seeking behaviours.
- To identify barriers to child health care-seeking behaviours.

## Methods and study settings

The study was conducted in the Somali regional state of Ethiopia from February to March 2018. An explorative study design using qualitative data collection tools was employed. The respondents were women of reproductive age and their partners, health extension workers (HEWs), health care providers and health administrators. Pre-tested, semi-structured FGD and individual interview guides and facility abstractions check list were used to explore factors including culture, religion or other root causes of low service utilization of reproductive, maternal, neonatal and child health (RMNCH) services and to assess the existing government and social structures. Additionally, other opportunities that had and potentially could have a role in increasing uptake of RMNCH services were assessed. To ensure representativeness, the region was categorized in to three areas according to settlement characteristics as Urban, Agro-pastoralist and pure pastoralist. From the three categories, two districts were randomly selected by Maternal, Child and newborn performance indicators from the Health Management Information System (HMIS) as high performing and low performing district; finally, one district from a high performing and the other one from a low performance district was included in the study. [Table 1]

**Table 1:** Sampling procedures used in selecting participants for data collection in deciphering barriers in reproductive, maternal and child health services in the Somali regional state, 2017.

S.No	Performances of the districts	Settlement characteristics			Total
		Urban	Agro-pastoralist	Pure Pastoralist	
1.	**High performing**	Kebridahar	Tuli-Guled	Shinille	3
2.	**Low performing**	Degehabour	Harshin	Warder	3
	**Total**	2	2	2	6

In the selection of the study sites, the heterogeneity of the area was taken into consideration and mothers or partners were selected during data collection by randomly selecting a household while walking in the selected district (random walk approach) and snowball sampling was also used to select mothers. Religious leaders, community leaders, health professionals (HEWs, midwifes, nurses and public health officers) were also selected from each study district for KII/IDI purposively.

To address demand side barriers in each district, 2 FGDs were conducted; one among mothers and the other among partners who were selected from the households. Furthermore, three IDI of mothers: family planning (FP) user, FP non-user and mothers with history of sick children in the last one month, one IDI of male partners who have children aged 0–12 months, two KII among religious leaders as well as one KII with community leaders were conducted. To address supply side barriers from each district, two FGDs were done; one was among health care providers and the other among HEWs in the selected district. In addition, five KII (health center head, district health office head, health center RMNCH head, health service providers from private clinic and pharmacy) were conducted at each district. Furthermore, KII among three zonal health office MCH heads and a Somali region health bureau MCH director head was conducted. Saturation of information was considered during our data collection and in total, 50 individual interviews (IDIs and KIIs), 17 FGDs, and 22 facility abstractions or observations were collected.

The Social Ecologic Model (SEM) was used for categorization. The data was written in a word processor and coded, categorized and analyzed using NVIVO version 11 software.

Ethical clearance was obtained from Saint Paul's Millennium Medical College (SPHMMC-IRBs). The team of data collectors obtained a permit for data collection from the Regional Health Bureaus, Zonal head Department (ZHD) and woreda health office before directly proceeding to data collection. Individual informed written consent was obtained before proceeding to the data collection.

## Results

**Fig 1:**
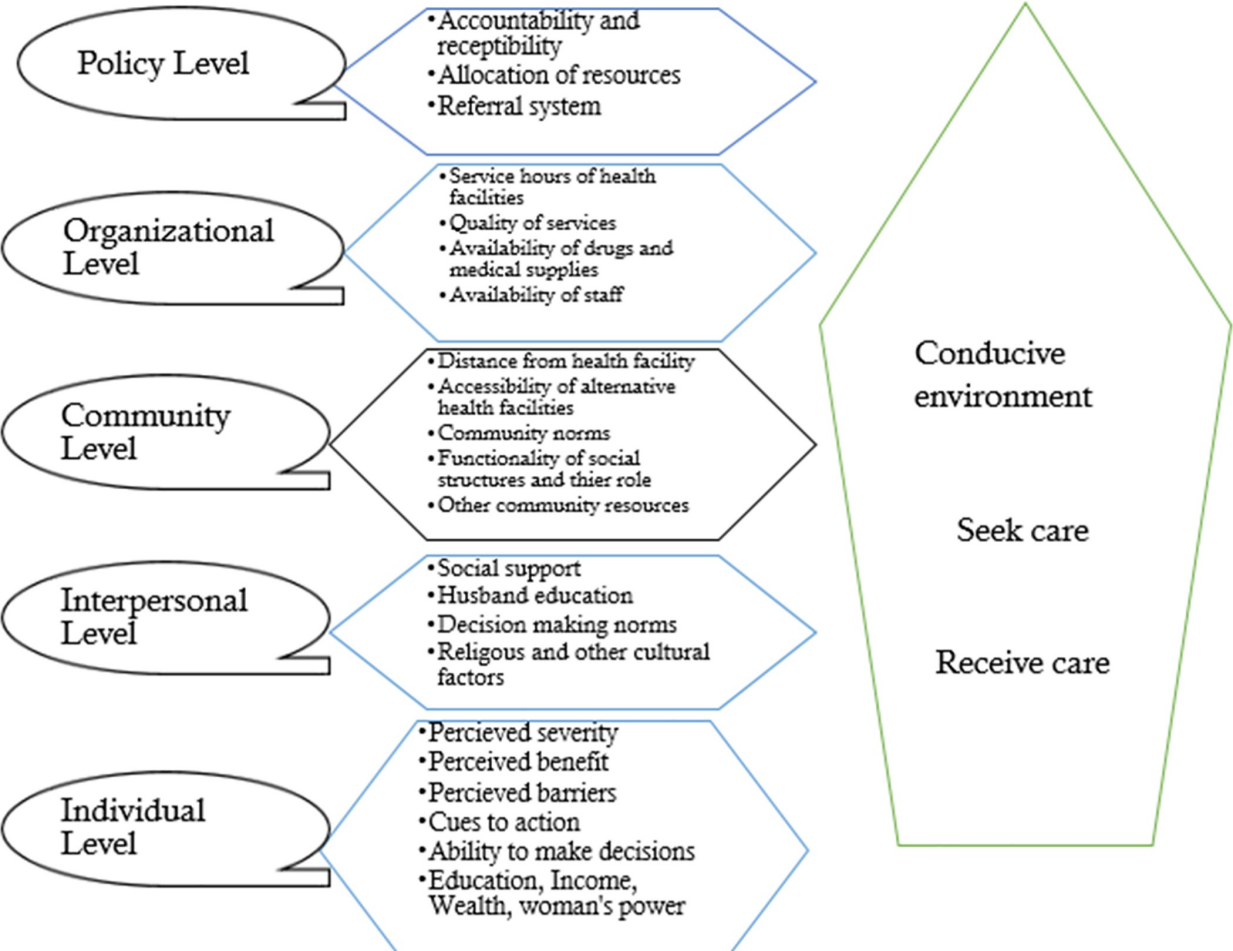
Social Ecological model used to decipher barriers in reproductive, maternal and child health services in the Somali regional state.

The result of this study described below was using the Social Ecological Model (Fig 1).

### Barriers at individual level

Uptake of RMNCH in the Somali region is majorly affected by residential area, socio-cultural and economic characteristics of the community.

For example, in urban settings people are aware of the availability of the service and have easy access to use the services, whereas in rural settings the conditions and lifestyle of the community may complicate health service seeking behavior in general and RMNCH uptake specifically. Additionally, literacy and having formal education played a big role as well. Moreover, lack of exposure to health education from health care providers, mass media/radio may also limit the knowledge of the community.

*“Even though the service is the same…there is a difference in utilization of the service between people who live in the urban and rural area*. *People who live in the town have better education and they utilize the services…*. *Mothers who come from rural areas visit the health facility very late*, *but those women who come from urban areas are educated and knowing something about the problem*, *so they come earlier*.*”* **FGD participant HCP, Kebridahar.**

Most of the respondents reported that due to poverty, the woman’s choice of health care was largely affected.

*“The mother can’t buy a banana for her child*. *It is a big problem…*. *if people have no money how they can go to health services*.*”* **FGD participant HEW, Fafan Zone**

Most of the respondents do not decide to visit the health facility early. Rather, they try every possible option they can at home and visit the health facility only after several home remedies have failed, which may sometimes cause irreversible disabilities to the mother or their baby.

*“Most of the time mothers came to the health facility if the labor is prolonged and they face any problem during delivery; apart from these conditions mothers always try to give birth at home”*. **KII participant Zonal health department head, Korahe Zone.**

In Somali and in other Muslim majority regions being assisted by a male attendant is not acceptable by the women; even the HEWs do not accept it. Mothers who receive services from female health care providers are more comfortable and satisfied with the services.

*“I always prefer the female health worker…*. *there is no problem getting service from a male health care provider*, *but since I am a woman*, *I prefer a female midwife*.” **IDI women, Kebridahar**

*“Yes*, *they don’t want to give birth assisted by a male health professional*.*”* **IDI HEW, Fafan zone**

### Barriers at interpersonal level

Almost all the female respondents replied that their role in the decision-making process is limited and that the power to make decisions is delegated to their husband. Similarly, a husband confirmed he is the one who decides on financial and health related issues. On the other hand, husbands are reported to be good at taking care of their family during times of sickness, but very restrictive regarding family planning.

*“The decision belongs to my husband because he is responsible for everything the family needs and does*. *So*, *it belongs to him*.*”* **IDI Mother, Kebridahar**

*“The decision belongs to me and I give them whatever they need but it does depend on why they go there and how sick they are*. *If it is something that requires extensive laboratory investigations*, *it would take a good amount of money but if it is simple*, *I would give them the money it would take and let them go*.*”* **IDI Husband, Warder**

Regarding the decision about the number of children to have, the husband is dominant over his wife. Both husbands and wives confirmed that most of the time they don’t decide (probably as they do not communicate clearly) about the number of children they will have, they believe the decision is from Allah.

“*We are people with faith*, *we have Islam and the towhead*. *So*, *there is nothing you can decide*, *it is sin*, *shame on you to say to me and my wife that for the next two years we won’t have a child*. *We can’t decide*, *that it is a sin on us so that is what we believe in*.*”* **IDI Husband, Kebridahar**

A similar answer was given by female respondent

*“Since we are Muslims*, *we rely on the will of God and we don’t believe in science that says if you do this you might not conceive*, *or you may conceive*.*”* **IDI Mother, Kebridahar**

Health care providers from urban high performing districts discussed that there are many challenges women face when they want to use some forms of health services such as family planning. Somali women are not supposed to use any form of family planning without consulting their husbands, and besides the influences of their husbands, they would also be stigmatized from the community. They also stated that since a woman’s husband expects her to become pregnant continuously, if she has any difficulty in conceiving or if he knows she is using family planning, he will divorce her immediately because he thinks she has killed his child.

*“That's right*, *in another location mothers may be able to use (family planning) without consulting their husbands but in Somali (state) women cannot use it…*. *their husband expects pregnancy from his wife just after one year of delivery*, *unless he monitors or follows her*, *she could secretly use a family planning method but if he confirms this*, *immediately he will divorce her*, *because her husband does not allow her to do any task without his permission*, *this is Muslim faith*.*”* **FGD HCP, Kebridahar.**

Regarding the husband’s support, all men respondents expressed positive support for their partners to go the health facility to get RMNCH, except for FP.

*“She has seen a health worker and I have encouraged her to see a health worker so that she knows about her health better*. *In addition to that*, *she has seen the health worker about 5–6 times while she was pregnant*.*”* **IDI Husband, Harshine**

A female respondent also replied about her husband’s support

*“He encourages me to see the health worker and to know a lot about my health and that of my child and also*, *he gives me all the money I need and without him*, *nothing would have been possible*.*”* **IDI Mother, Tuliguled**

Word of mouth is very influential in the Somali region and effective when it comes to information sharing. This can either positively or negatively affect the RMNCH service utilization. Mothers even trust the information from their non-Somali neighborhoods who have used the services and have finally accepted and used the services.

*“Mothers and children who become healthy after they get health care services are helpful for teaching others*.*”* **FGD HEWs Degehabour**.

*“There was a non-Somali woman at my neighbors and she told me that she uses such services*, *and also before I have heard about family planning from health extension workers*, *and I start to take tablets by going to the hospital every three months*.*”* **IDI FP user Shinille**

### Barriers at community level

In the Somali region, traditional birth attendants (TBA) are still preferred for delivery services. Also, in some areas of the region the TBAs are being used by the public health facilities as institutional delivery service promoters through using incentives when TBAs link or bring a pregnant or laboring mother with them to the public health facility.

*“Many people prefer the traditional service*, *especially for delivery services*, *but it is impossible to get a medicine*, *for example*, *for excessive bleeding with traditional services and the procedures also not clean*.*”* **FGD HCW, Harshine**

“S*omalis say you should go to traditional healers and religious healers if someone gets sick and after he spends his money there*, *he should be taken to the health facility*.*”* **FGD HCW, Harshine**

TBAs are treated differently in different zones within the region; for example, TBAs from low performing urban areas will be paid for the service they provide and work with health care providers. But in other parts, TBAs from similar performance areas (low performing) are not paid for the services they provide, and they are discouraged and not interested to work with health care providers because of lack of incentives.

*“They (TBAs) give health education for the mothers*. *They advise mothers to give birth at a health facility*. *They are also paid for the service they provide*.*”* ***FGD* HCP, Degahabour**

*“There are traditional birth attendants for delivery called (TBA)*. *Those individuals do not cause any harm to the community and we tried to work together with them*. *They are not interested to work with us*. *They don’t have a salary*. *They are not trained*. *They are delivering by moving inside the city*.*”* **KII head of health center, Warder**

Even though there was a deep-rooted belief that the majority of the Somali community prefer TBAs to modern medicine or trained birth attendants, some women disliked going to TBAs as they were concerned that she would transmit blood-borne infections from infected mothers to uninfected newborns as the TBAs use unsterile scissors and blades for delivery.

*“I don’t like the traditional birth attendants because you might die of bleeding or they use scissors and razors they used for another person which could transmit diseases*.*”* **IDI Women, Degahabour.**

The community discussed that birth spacing has a great advantage for both the mother and her baby and they believed that if there is no gap in between births, there might be many medical problems the mother and baby face. They recommended that there should be about a two-year gap in between the consecutive births. The interesting issue is that the religion also supports birth spacing for at least two years before the second birth. Likewise, they reported birth spacing creates more time for the baby to breast feed and breast feeding minimizes the risk associated with repeated delivery.

*“Child birth spacing helps the child to have more time for breast feeding and it prevents the risk of having health problems from frequent delivery*.*”* **Family planning user, Wardar**

*“I believe in spacing between children and I recommend to space for about two years because it is what our religion recommends us to do*.*”* **Family planning user,** Kebridahar

### Organizational (structural) factors

Under the same circumstances some women use the services more than the others. This shows that there are factors other than the health care service characteristics that influence the use of maternal and reproductive health services.

*“……mothers come from any rural and urban area for antenatal care service …*. *Even they have knowledge about follow up*, *knowing their disease*, *knowing their drugs for treatment but ultimately*, *they give birth at home for a strange reason”* **FGD HCP, Kebridahar**

The participants reported that the health need of the community is beyond the service the health post is intended to provide, so if they cannot get new services, they are not willing to subsequently use the services from the HEWs.

*“The community says the health post is too narrow and there are no laboratory services so why should we came to the health post and what will you do for us*? *… The community says no medication and you do not take urine or blood for laboratory tests*. *You can do nothing when we come to the health facility*.*”* **IDI District health department head, Kebridahar.**

*“At the health post level*, *communities are not coming to get these services*, *because they are going to hospital to get better treatment and care in our city… the community starts requesting services when they need it*.*”* **FDG HCP, Degehabour**

The preference to use of private hospitals rather than public hospitals is because the mother needs privacy and well-ventilated rooms that they do not get from the government hospitals.

*“That is their choice*. *For example*, *when you look at the hospital level*, *they prefer to go to a private hospital*. *It depends on your approach*. *Because there is no crowd there*. *They get treatment in a relaxed atmosphere*, *with ventilated air*. *There are some patients who refused to get treatment*. *You take consent and send them where they want to go to*. *You can understand when you observe nearby*.*”* **FGD HCP, Shinille**

HEWs reported that their health post has no comfortable area where women psychologically would want to give birth due to lack of water and hygiene. Most of the health posts do not have a water supply, so they cannot provide clean delivery services there. Mothers prefer not to go to such a health post for delivery, preferring home delivery instead.

*“Most mothers deliver at home because there is no water*, *sometimes no surgical gloves and an unclean area in the health post*.*”* **FGD HEW, Warder.**

Most of the mothers who gave birth at the health facility were happy and comfortable with the institutional delivery services.

“*I was very happy because my baby was 4KG*. *So*, *without a health care providers’ assistance it was difficult to give birth*.*”* ***FP* user mother, Tuliguled.**

In places where there was an option of both hospitals and other primary health care units (PCHU), the health care providers stated that the mother did not use the delivery services for fear of invasive procedures like instrumental delivery and caesarian section. The few individuals who are using institutional delivery prefer to go to hospital rather than using the nearby health post or health center. There was self-referral to the hospital by the community for perceived better care. But in other places, especially the low performing pastoralist region (Dolo), major barriers raised were absent or nonfunctional health facilities, a shortage of supplies, lack of support and response from the regional health bureau, an unclear bureaucratic system from the region to the district and a shortage of water, electricity and transportation. Furthermore, the HEWs are not always available at the health post as almost all their time is spent providing outreach community services.

*“There is no infrastructure… we are not getting enough supplies from the regional (offices)*. *When we request something from the Woreda*, *they say it is from region*, *and when we asked from the region*, *they said the Woreda is responsible for such type of logistics*. *I don’t know*, *nothing is coming in each year*. *When the budget was announced we knew there is a budget allocation for the health post*. *But we didn’t see anything related to these issues…*. *I don’t know about these issues*. *We don’t have any support except for some NGOs*. *There is no governmental support for material related support*. *NGOs are supporting us related to child health*. *WHO is working together with us with EPI”* **KII Warder HC head.**

The main reason why women prefer home delivery and do not like to go to the health facility for delivery is that most of the health posts do not have a water supply, so they cannot provide clean delivery services there for mothers.

*“Majority of the health posts have no water supply and cleaner*. *Hence there are no clean delivery services provision*. *Therefore*, *pregnant mothers do not want to give birth at the health post rather they prefer to give birth at home*. *So*, *it is not possible to attend delivery with dirty water”*. **FGD HEW, Kebridahar.**

At the rural level the postnatal care (PNC) service utilization is low. Even compared to the institutional delivery services, the PNC services utilization is very low in Somali region. The perception of not visiting a health facility when feeling well was the main cause for not using postnatal care services by the community.

*“Mothers feeling healthy during the postnatal cared period was the main reason for not using PNC services*.*”* **FP non-user husband, kebridar.**

### Roads and communication services (infrastructure)

The difficult nature of the road and inaccessibility of mobile cell phones is a challenge in the service delivery system.

*“…But according to our district*, *distant kebeles have problems concerning arriving on time*. *This is sometimes due to lack of mobile and network*, *but others come despite these problems”*. **KII MCH head, Fafan Zone**

The problem is not only financial, but sometimes women may not find a car and end up coming on foot from very distant areas (about 80 km from the health facility). Often, when they arrive, they have life threatening complications.

*“The mother cannot come if the car is not available*. *For instance*, *clients from some districts like*, *Degamire*, *Bergode and Aware health centers come to our hospital*. *Because of the lack of the services in these health centers*, *they come to our hospital after developing complications*.*”* **KII MCH head, Fafan Zone.**

### Distance

There were three types of distances discussed by participants. The first distance discussed was distance between the households. Because of the different settlement characteristics, the distance between one household to the next household may reach from 2–5 km. The second distance discussed was the distance between health facilities (health centers, Health posts etc.) and the households of the residents may reach about 80km. and the third distance discussed was the distance between the health facilities may far from each other (30–70 km). Participants exhaustively discussed that distance is a major barrier to utilize the available services even if they have the capacity to use the services. The distance from one household to the next household was found to be challenging as it is difficult for one health extension worker to reach each household. Where the other may make difficulty in using the health centers and hospitals either for routine or for referral services.

*“During the outreach programs it is a must to go at least 2 km to get from one household to the other household here in Somali region; that means the community is dispersed*. *There is no car or transportation system*. *Due to this*, *one health extension worker feels fed-up after 1 or 2 household visits in the morning and the weather conditions are too hot in the afternoon time and it is difficult to work*,*”* **FGD HEWs, Harshine.**

*“…*. *They are located far away from us*. *They are located 30km far away from each other*. *In some areas they are 70km far away from each other*.” **KII Warder Health center head.**

Most respondents discussed that lack of transportation was challenging to pregnant women to get health services at health facilities because of this and the unavailability of ambulance services they prefer to give birth at home. Even though health care providers plan to serve the far-off areas, because of shortage of transportation they cannot. Health care providers also wished to have other alternatives for transportation services like ‘Bajaj’ or motorcycles since they have a shortage of ambulances.

In the region maternal delivery services are provided free of cost. The other services which are given at the health facility are affordable in terms of cost. In addition, there is a trend in the Somali community for community members to support each other or help those in need. Some members of the community utilize the health services of the traditional healers because the service is nearby and therefore cheaper.

*“I can get the health service easily if I will go to the health facility and it is free of charge but the only cost, I pay is that of the taxi*.*” **IDI Mother with sick child, Harshine***

*“As I have seen the community members easily speak their problem to traditional healers and also*, *I think the cost is cheaper than the health institution or it may be freely given.” **IDI Mother with sick child, Warder***

In some public hospitals there are maternity waiting homes, which are funded by UNICEF, in which pregnant women can stay up to 2 months or until she gives birth. During their stay she has free shelter and foods such as milk, biscuits and ‘temir’, also funded by UNICEF.

*“……the mothers stay at the maternity waiting home till they deliver*. *For instance, if she is in the 8th month of pregnancy we admit her until she delivers…. the fee is covered by UNICEF. We asked her what she wants to eat. They have milk and biscuits, temir also there.” **KII, MCH coordinator Degehabour hospital***

The participants reported there was a shortage of trained human resources in the health facility and because of this the health post were sending back clients without offering health services, especially in low performing pastoralist areas. One of the issues is that those few professionals who have been trained move from the rural setting to the better health facilities in urban areas. Even then, those who are trained do not always provide the services because of religious and cultural issues; the best example being family planning. Apart from the shortage of health care providers, TBA themselves are reported to be very few in some locations (low performing pastoralist area), On the other hand, health care providers who work in remote areas are in need of incentives, yet sometimes they do not even get their salary on time.

*“We don’t have sufficient trained professionals*, *especially nurses”*. **FGD HCP, Kebridahar Hospital**

*“The people are coming but we send them back without offering the services*. *We don’t have trained man power and materials*. *When we are saying that we offer abortion*, *we are not all trained*.*”* **FGD HCP, Shinile**

*“So*, *for those all health centers we are offering basic emergency and obstetric care (BEmONC) for mothers and child health issues”*. **Head DHO, Kebridahar**

## Discussion

RMNCH service-seeking behavior of the Somali region community was affected in a major way by residential area and the socio-cultural and economic characteristics of the community. For instance, in urban settings, people are aware of the availability of the service and have easy access to use services because they have some exposure to health information either through media, health care providers or through their peers. In rural settings the living conditions and lifestyle of the community caused low exposure to media, low or no exposure to formal education complicated health service seeking behavior of the residents. Due to this, rural residents face a greater challenge in using RMNCH services as most of them do not visit the health facility on time, but rather use other possible options which were acceptable, accessible and affordable to them. For example, residents can use home remedies or TBAS, otherwise they visit health facility only after everything else has failed, which may sometimes cause irreversible disabilities to the mother and their baby. This finding is supported by similar other studies [2–5].

This research described how, for educated Somalis, the preference was not for quantity of children but quality of life for children and the family was given priority. For uneducated Somalis, the quantity of children matters as women with many children are respected by their peers and the children were also seen as an asset for the family. This study was supported by another research project that showed that for an educated man, having a large number of children was not considered as the source of wealth, rather the family learns to live within its own limited economic resources and start giving more attention to the quality of life for their children [24].

In Somali and in other Muslim majority regions, being assisted by a male attendant is perceived unacceptable by women; even the HEWs do not accept it. This study showed that except for a few mothers who have no specific sex preference, almost all mothers preferred to get maternal health care services from some female health care providers. Mothers who were attended by a male attendant would not be satisfied with the services she received. She may not visit the facility again for maternity services and may also influence others to not go to the facility.

This finding was supported by other studies in Ethiopia and Egypt who reported that in the Muslim religion, males are not permitted to touch women so they have fear delivering at hospital with male professionals [6, 7]. In addition, published papers from Nigeria depict the leading reason for long acting contraception non-use was conflict with religious or cultural beliefs [8], as do similar studies [2, 9–11].

In the Somali region, men are the leaders of the family and make most decisions including health and health related issues. They will take care of their family at all levels and support all other related health services except family planning. Using family planning is seen as taboo in the region. Some other obstacles to using FP were women fearing that their husband can marry other women if they use FP (in a polygamous context), fear of side effects from contraception and fear of stigma from the community. But there were differences in decision making processes among educated married men and non-educated married men. For example, educated men may allow women to use family planning services and women from these families have a wide range of options. Customary practices for men and women are different in the community. A similar study by Beekle illustrated that current contraceptive usage was found to be strongly associated with spousal discussion about family planning. [7, 11, 25]. Research from Nigeria found that 80% of women reported they do not use FP because of feared side effects [26]. Other common reasons included no perceived need for a longer-acting method, belief that longer-acting methods cause infertility and partner objection [8].

The findings that there were too few health professionals in the low performing areas and many who were trained would move to urban areas is supported by study funded by DFID (2006). The shortage of trained health care providers in rural settings acted as a barrier to seeking health services in the region which could be generalized as availability, quality and affordability of maternal health care services greatly influences the use of the services by women [27, 28].

The other barriers discussed by the Somali region community were that regardless of the cost, the community prefers private health facilities to public due to lack of privacy, overcrowding, mistreatment by the health care providers, unclean environment at public health facilities and long waiting times. The study is supported by other similar studies that reported that when the quality of services in public clinics is perceived as low, even with low user fees, individuals, including the poor, may prefer to go to a private provider for care [10, 29, 30].

## Conclusion

The poor health-seeking behavior of the community in this region is multi-factorial. socio-demographic and economic factors like culture, religion, preference for female care providers low socio economic characteristics, the deep-rooted patriarchal system in the region, the role of word of mouth, trusting traditional customs and practices, dysfunctional bureaucratic system at the regional level, the shortage of medical supplies, human resources, lack of supportive supervision, and a shortage of water and electricity at the health facility, un clean service delivery environment are significant barriers to health-seeking behaviors for the community.

## Recommendations

One solution may be for the local government and the Federal Ministry of Health to consider upgrading the current level of TBA to becoming community midwives and equipping the facilities with medical supplies and trained human resources. At the community level, local leaders can be trained on some key RMNCH topics, particularly family planning or family spacing, to train the community. Issues of transport and distance also need to be resolved by alternative means of travel. This will lead to a change in the community’s RMNCH service seeking behaviour and can also increase the health service utilization in the region.

## Supporting information

S1 File HSB interview guide. (RAR)
